# Triple-Component Lung Carcinoma Diagnosed After Nondiagnostic EBUS: A Case Report

**DOI:** 10.7759/cureus.112412

**Published:** 2026-07-10

**Authors:** William Le, Megana Tirupathi, Luke Oh, Michael Diaz, Sreelatha Tirupathi

**Affiliations:** 1 Internal Medicine, Nova Southeastern University Dr. Kiran C. Patel College Of Osteopathic Medicine, Clearwater, USA; 2 Internal Medicine, Idaho College of Osteopathic Medicine, Meridian, USA; 3 Internal Medicine, Touro College of Osteopathic Medicine, Middletown, USA; 4 Oncology, Florida Cancer Specialists and Research Institute, St. Petersburg, USA; 5 Internal Medicine, All Florida Family Care Inc, St. Petersburg, USA

**Keywords:** adenocarcinoma lung, combined small-cell lung carcinoma (c-sclc), ebus-tbna, large-cell neuroendocrine carcinoma, mediastinal lymphadenopathy, nondiagnostic biopsy, small-cell lung cancer, surgical lung biopsy, triple-component carcinoma, tumor heterogeneity

## Abstract

Combined lung carcinomas containing both non-small cell and high-grade neuroendocrine components are rare and diagnostically challenging due to intratumoral heterogeneity. We report a 61-year-old never-smoking woman with pleuritic chest discomfort and chronic cough. Computed tomography (CT) and positron emission tomography (PET)/CT demonstrated a left upper-lobe pulmonary nodule with intensely FDG-avid hilar and mediastinal lymphadenopathy. Endobronchial ultrasound-guided transbronchial needle aspiration (EBUS-TBNA) of FDG-avid lymph nodes was nondiagnostic. Given persistent clinical and radiologic concern for malignancy, the patient underwent video-assisted thoracoscopic surgery (VATS) for definitive diagnosis. Pathology revealed a rare triple-component malignancy composed of adenocarcinoma, large-cell neuroendocrine carcinoma, and small-cell lung carcinoma, with metastatic neuroendocrine carcinoma involving mediastinal lymph nodes and pleura, consistent with stage IVA disease. The patient was subsequently treated with platinum-etoposide-based systemic therapy, with immunotherapy planned as part of her treatment course. This case highlights that nondiagnostic minimally invasive sampling does not exclude malignancy and underscores the importance of radiologic-pathologic concordance, awareness of sampling limitations, and timely escalation to additional tissue acquisition, including surgical biopsy when appropriate.

## Introduction

Combined lung carcinoma is a heterogeneous group of tumors most commonly described as combined small cell lung cancer (cSCLC), in which small cell lung carcinoma (SCLC) coexists with a non-small cell lung carcinoma (NSCLC) component [1-3]. These mixed-histology tumors carry important diagnostic and therapeutic implications, as the presence of high-grade neuroendocrine components, such as SCLC or large-cell neuroendocrine carcinoma (LCNEC), typically drives prognosis and necessitates treatment according to the most aggressive lineage [4-7].

Accurate diagnosis of combined lung carcinomas is challenging, particularly in small biopsy specimens. Tumor heterogeneity, coupled with molecular and phenotypic overlap between SCLC and LCNEC, increases the risk of sampling error and misclassification on limited cytologic specimens [7-9]. Consequently, nondiagnostic or misleading biopsy results may delay initiation of appropriate platinum-based systemic therapy, which is critical for high-grade neuroendocrine malignancies [4, 5]. Recognition of combined histology is therefore essential, as therapeutic management is generally guided by the neuroendocrine component rather than the accompanying NSCLC subtype.

Triple-component tumors containing adenocarcinoma, SCLC, and LCNEC are exceptionally rare, with only one directly comparable prior case identified in the literature [3]. Such profound intratumoral heterogeneity further complicates diagnosis, as focal neuroendocrine components may be missed on minimally invasive sampling [6, 8, 9]. In this context, we report an additional case of triple-component lung carcinoma consisting of adenocarcinoma, LCNEC, and SCLC, diagnosed only after surgical biopsy despite suspicious clinical and CT findings, including an upper-lobe pulmonary nodule with associated hilar and mediastinal lymphadenopathy, supportive positron emission tomography/computed tomography (PET/CT) findings, and nondiagnostic endobronchial ultrasound-guided transbronchial needle aspiration (EBUS-TBNA) [10, 11].

## Case presentation

A 61-year-old never-smoking woman with chronic obstructive pulmonary disease diagnosed on prior spirometry, hypertension, hyperlipidemia, osteoarthritis, migraines, major depressive disorder, and a remote ischemic stroke presented with pleuritic chest discomfort precipitated by coughing. She reported a chronic cough for more than 20 years, previously attributed to chronic obstructive pulmonary disease despite her lack of smoking history. She denied hemoptysis, dyspnea, fever, or unintentional weight loss but endorsed intermittent drenching night sweats for several months.

**Figure 1:**
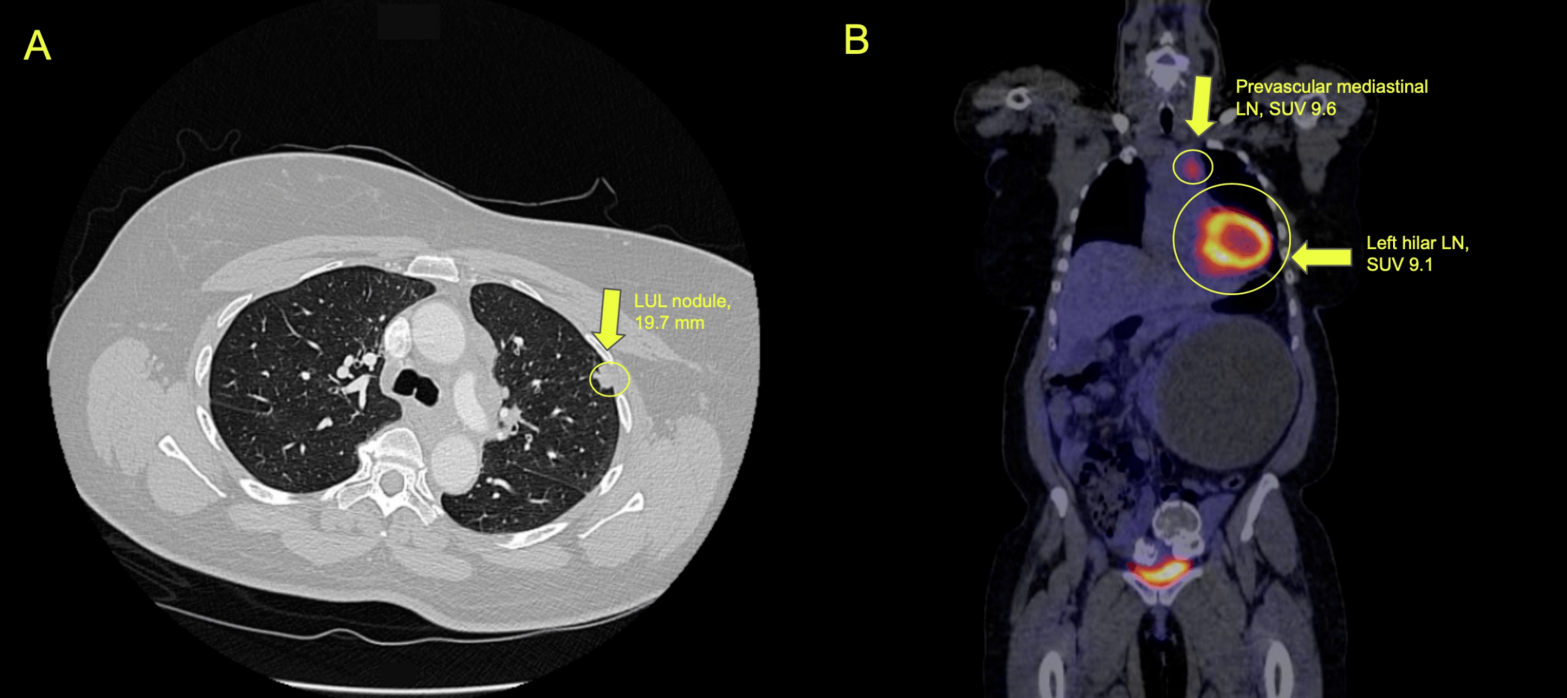
Chest computed tomography (CT) and positron emission tomography (PET)/CT demonstrating the primary left upper-lobe pulmonary lesion and associated FDG-avid nodal disease preceding nondiagnostic sampling. (A) Axial lung-window CT demonstrating a lobulated solid nodule in the left upper lobe measuring approximately 19.7 mm. (B) Coronal fused PET/CT demonstrating intensely FDG-avid left hilar lymphadenopathy with SUV 9.1 and prevascular mediastinal lymphadenopathy with SUV 9.6. The primary left upper-lobe lesion demonstrated comparatively mild FDG uptake with SUV 2.0. Annotations identify the primary pulmonary lesion and FDG-avid nodal regions. CT: computed tomography; PET: positron emission tomography.

She was initially treated empirically for presumed bronchitis. Chest radiography was unremarkable; however, persistent pleuritic symptoms prompted contrast-enhanced CT of the chest, which demonstrated a 19.7-mm lobulated solid nodule in the left upper lobe with an associated left hilar nodal mass and mildly enlarged mediastinal lymph nodes, raising concern for malignancy. Given these suspicious CT findings, PET/CT was subsequently obtained before tissue diagnosis to further characterize metabolic activity, evaluate for nodal or distant disease, and guide the safest and highest-yield tissue sampling approach. PET/CT revealed intensely FDG-avid left hilar (SUV 9.1) and prevascular mediastinal (SUV 9.6) lymph nodes, with comparatively mild FDG uptake in the left upper-lobe lesion (SUV 2.0). A mildly FDG-avid precarinal lymph node was also noted. Several renal cystic lesions were identified but demonstrated no FDG avidity (Figure 1). Because radiology was unable to perform a CT-guided needle biopsy of the pulmonary lesion, the patient was referred for bronchoscopic evaluation with EBUS-TBNA, which was negative for malignancy, tuberculosis, fungal infection, and lymphoma. Despite these nondiagnostic findings, the patient’s clinical presentation and persistent clinical and radiologic concern for malignancy prompted referral to cardiothoracic surgery for video-assisted thoracoscopic left upper-lobe wedge resection and lymph node dissection.

EBUS-TBNA of the FDG-avid mediastinal and hilar lymph nodes yielded lymphocytes and macrophages without evidence of malignancy, granulomatous inflammation, or fungal or mycobacterial organisms. Cytologic evaluation was nondiagnostic. Despite these findings, clinical and radiologic concern for malignancy remained high. Percutaneous biopsy of the pulmonary lesion was deemed unsafe by interventional radiology because of its proximity to vascular structures. Because EBUS-TBNA was nondiagnostic and CT-guided needle biopsy could not be performed, multidisciplinary consensus favored video-assisted thoracoscopic surgery (VATS) with wedge resection of the left upper-lobe lesion and mediastinal lymph node dissection as a diagnostic surgical biopsy to obtain a definitive tissue diagnosis and pathologic assessment of nodal disease.

**Figure 2:**
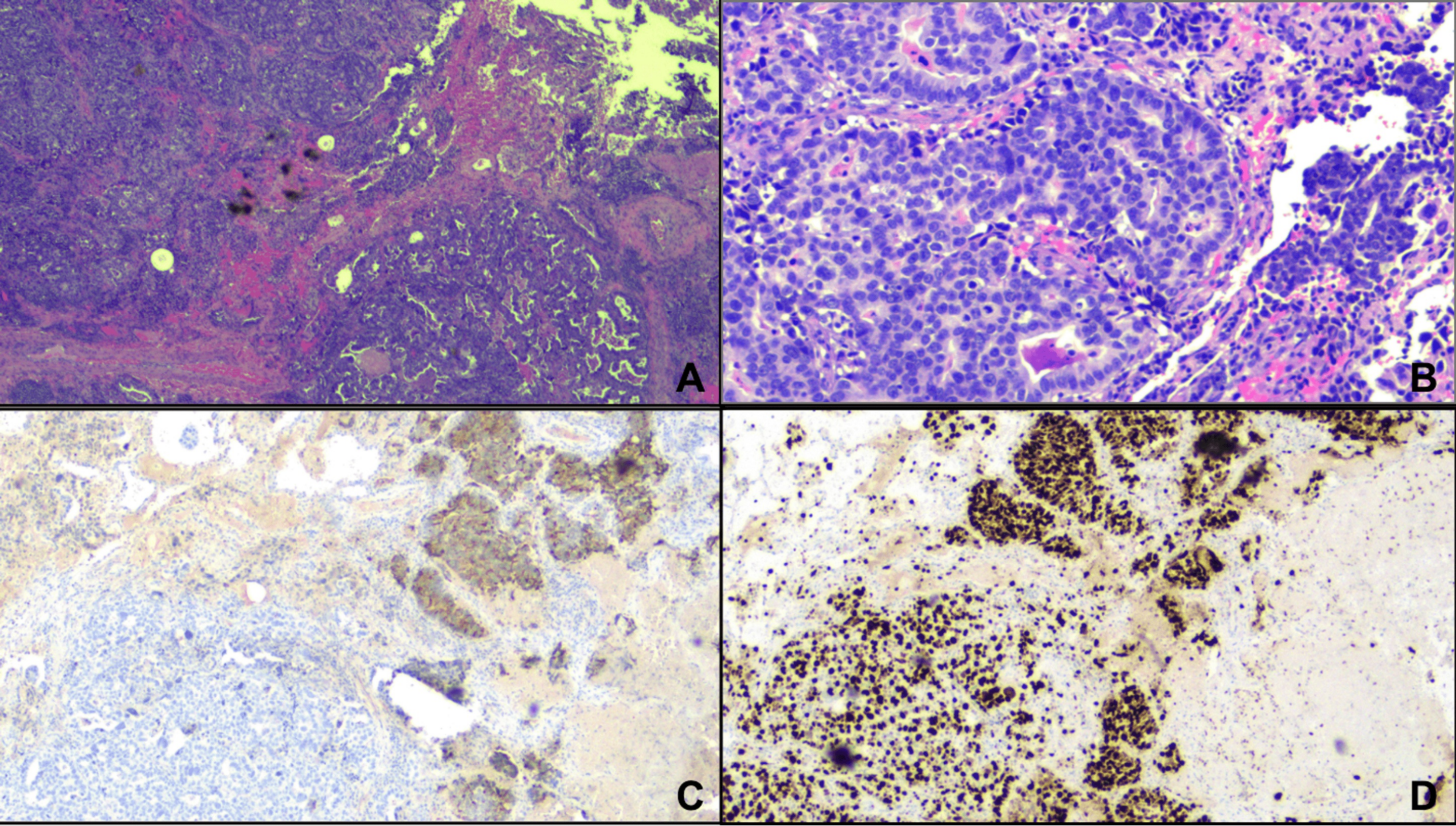
Histopathologic features demonstrating marked intratumoral heterogeneity in a triple-component lung carcinoma. (A) Low-power hematoxylin and eosin (H&E) stain demonstrating invasive acinar adenocarcinoma (lower right) adjacent to high-grade neuroendocrine carcinoma (upper left). (B) Higher-power H&E stain demonstrating high-grade neuroendocrine carcinoma with organoid architecture and granular chromatin. (C) Chromogranin immunohistochemistry demonstrating strong positivity in the neuroendocrine carcinoma component (upper right) with the absence of staining in the adenocarcinoma component (lower left). (D) Ki-67 immunohistochemistry demonstrating a markedly elevated proliferative index (~90%) in the neuroendocrine carcinoma component (upper right), with substantially lower proliferative activity in the adenocarcinoma component (lower left).

Pathologic examination revealed a rare combined malignancy composed of invasive acinar adenocarcinoma, LCNEC, and SCLC within a single 1.8-cm tumor (Figure 2). LCNEC was distinguished from SCLC by larger cell size, prominent nucleoli, organoid growth patterns, and lower nuclear-to-cytoplasmic ratios, whereas the SCLC component demonstrated smaller cells with scant cytoplasm, nuclear molding, and a markedly elevated proliferative index.

Immunohistochemistry demonstrated CK7, TTF-1, and Napsin A positivity in the adenocarcinoma component, with synaptophysin, chromogranin, and CD56 positivity in the neuroendocrine components. The Ki-67 labeling index approached 90% in the SCLC component, with substantially lower proliferative activity in the adenocarcinoma component. Visceral pleural invasion and spread through air spaces were present. Surgical margins were negative, and no lymphovascular invasion was identified.

Metastatic neuroendocrine carcinoma was identified in a station 7 mediastinal lymph node and in pleural biopsies, establishing AJCC 8th edition stage IVA (pT2aN2M1a) disease, with the M1a classification based on pleural involvement.

The postoperative course was uncomplicated. The patient was referred to medical and radiation oncology and initiated on intravenous systemic therapy with carboplatin and etoposide administered every three weeks in combination with trilaciclib, with atezolizumab planned outside the period of concurrent radiation. A port catheter was placed to facilitate chemotherapy administration.

Radiation oncology recommended intensity-modulated radiation therapy to a total dose of 60 Gy in 30 fractions delivered over approximately six weeks, targeting the left upper lobe lesion, pleura, mediastinum, and hilar lymph nodes. Brain magnetic resonance imaging demonstrated no evidence of intracranial metastases.

## Discussion

Combined lung carcinomas containing both NSCLC and high-grade neuroendocrine carcinoma are rare and clinically aggressive entities. Most reported cases involve cSCLC, in which SCLC coexists with an adenocarcinoma or squamous component [1, 2]. Tumors containing three distinct malignant lineages, including adenocarcinoma, LCNEC, and SCLC, are exceptionally uncommon. A comparison of the present case with the closest previously reported triple-component lung carcinoma containing adenocarcinoma, SCLC, and LCNEC is provided in Table 1. To our knowledge, only one directly comparable prior case has been reported, underscoring the rarity of this tumor combination and the diagnostic challenge posed by marked intratumoral heterogeneity [3].

**Table 1:** Comparison of the present case with the closest previously reported triple-component lung carcinoma containing adenocarcinoma, SCLC, and LCNEC. SCLC: small cell lung carcinoma; LCNEC: large-cell neuroendocrine carcinoma.

Study	Age/Sex	Tumor location	Histologic components	Diagnostic method	Stage/metastatic disease	Key comparison with the present case
Fellegara et al. [3]	69/M	Right upper lobe	Acinar adenocarcinoma + SCLC + LCNEC	Surgical/pathologic examination	NR	Closest previously reported case with the same three core histologic components. Molecular analysis supported a shared genetic profile across tumor components.
Present case	61/F	Left upper lobe	Invasive acinar adenocarcinoma + LCNEC + SCLC	VATS wedge resection and mediastinal lymph node dissection after nondiagnostic EBUS-TBNA	Stage IVA, pT2aN2M1a, with pleural involvement	Demonstrates the same rare triple-component combination, diagnosed only after surgical biopsy despite suspicious clinical/radiologic findings and nondiagnostic EBUS-TBNA.

High-grade neuroendocrine lung carcinomas, including LCNEC and SCLC, are characterized by rapid proliferation, early metastatic spread, and poor prognosis [4, 5, 7]. Although LCNEC is formally classified within the NSCLC category, its biologic behavior and clinical outcomes more closely resemble those of SCLC, particularly in tumors containing mixed neuroendocrine components [4, 5, 8]. Consequently, therapeutic decision-making is typically guided by the most aggressive histologic component rather than the accompanying NSCLC subtype [4, 5, 7].

Accurate diagnosis of combined lung carcinomas is frequently hindered by sampling limitations. EBUS-TBNA is widely used for mediastinal staging because of its favorable safety profile and high reported sensitivity; however, false-negative and nondiagnostic results are well documented [8-11]. In the present case, EBUS-TBNA of FDG-avid mediastinal and hilar lymph nodes yielded only benign lymphocytes and macrophages despite persistent clinical and radiologic concern for malignancy. Several mechanisms may explain this discordance, including sampling error, inadequate representation of malignant areas, focal or uneven distribution of neuroendocrine metastases, and marked tumor heterogeneity. These limitations are particularly relevant in combined tumors, in which aggressive high-grade neuroendocrine components may be focal and therefore missed by limited tissue sampling [6, 8, 9].

This case illustrates the importance of radiologic-pathologic concordance in the evaluation of suspicious pulmonary nodules with associated hilar or mediastinal lymphadenopathy. In patients with concerning CT findings, PET/CT may help further characterize metabolic activity, identify nodal or distant disease, and guide the most appropriate biopsy target; however, PET/CT should be interpreted as supportive rather than definitive. When CT findings and clinical features remain concerning despite nondiagnostic EBUS-TBNA, clinicians should reassess whether the sampled lymph nodes corresponded to the most suspicious radiographic abnormalities, evaluate the adequacy of tissue sampling, and determine whether the primary lesion or nodal disease is accessible by an alternative biopsy approach.

When less invasive sampling is nondiagnostic and persistent clinical or radiologic concern remains high, escalation to additional tissue acquisition should be considered. Options may include repeat bronchoscopic sampling, CT-guided biopsy, mediastinoscopy, or surgical biopsy depending on lesion location, nodal accessibility, procedural risk, and the likelihood that additional tissue will alter management. In the present case, CT-guided needle biopsy was not feasible because of proximity to vascular structures, and multidisciplinary consensus favored VATS wedge resection with mediastinal lymph node dissection as a diagnostic surgical biopsy to obtain definitive tissue diagnosis and pathologic assessment of nodal disease.

Accurate histologic classification has substantial therapeutic implications. While isolated adenocarcinoma may be managed using NSCLC-directed regimens, the presence of high-grade neuroendocrine carcinoma necessitates treatment strategies aligned with SCLC or LCNEC protocols [4, 5, 7]. Platinum-based chemotherapy with etoposide remains the backbone of treatment, with increasing incorporation of immune checkpoint inhibitors and radiation therapy in selected patients [4, 5, 7, 8]. Recognition of combined and mixed-histology tumors is therefore critical to avoid undertreatment and delays in care.

Overall, this case highlights the diagnostic limitations of minimally invasive sampling in highly heterogeneous lung carcinomas and emphasizes the need for continued diagnostic evaluation when clinical and radiologic findings remain suspicious despite nondiagnostic EBUS-TBNA. Early recognition of radiologic-pathologic discordance, awareness of sampling limitations, and timely escalation to definitive tissue diagnosis are essential for accurate classification and appropriate treatment planning.

## Conclusions

This case highlights the diagnostic limitations of minimally invasive sampling in heterogeneous lung malignancies. In patients with persistent clinical and radiologic concern for malignancy, nondiagnostic EBUS-TBNA should be interpreted in the broader context of radiologic-pathologic concordance, sampling adequacy, and tumor heterogeneity. Combined lung carcinomas may contain focal high-grade neuroendocrine components that can be missed by limited tissue sampling. When less invasive biopsy approaches are nondiagnostic or not feasible, escalation to additional tissue acquisition, including surgical biopsy, should be considered to establish an accurate diagnosis and guide timely treatment. Recognition of mixed-histology tumors is critical, as management is often driven by the most aggressive component, particularly high-grade neuroendocrine carcinoma.
